# What Healthcare Workers Told Us about Working through the COVID-19 Pandemic: A Qualitative Analysis of Digital Audio Stories

**DOI:** 10.1155/2023/8858475

**Published:** 2023-07-12

**Authors:** Claudia Grobbel, Lori Lackman Zeman, Ramin Homayouni, Sujoy Roy, Floyd E. Kenney, Elie Mulhem

**Affiliations:** ^1^School of Nursing, Oakland University, Rochester, MI, USA; ^2^Department of Family Medicine, Beaumont Health, Troy, MI, USA; ^3^Department of Family Medicine and Community Health, Oakland University William Beaumont School of Medicine, Rochester, MI, USA; ^4^Department of Foundational Medical Studies, Oakland University William Beaumont School of Medicine, Rochester, MI, USA; ^5^Oakland University William Beaumont School of Medicine, Rochester, MI, USA

## Abstract

**Aims:**

The purpose of this article is to share the qualitative results of digital stories from 21 healthcare workers during the second wave of the pandemic and compare them with stories analyzed during the first wave.

**Background:**

Everyone has personal COVID-19 experiences and memories. Yet, the literature is lacking in how the stories from the healthcare team during the pandemic affected the individual healthcare worker at that time.

**Methods:**

A descriptive qualitative study analyzing digital stories was conducted during the second wave of the pandemic of a large Midwest healthcare system.

**Results:**

Twenty-one audio stories were analyzed. Four themes revealed were negative emotional response/impact, feelings/statements of optimism and hope, imposed or changing role expectations, and leadership/administration concerns. These themes aligned with the first wave analysis.

**Conclusions:**

Healthcare workers during COVID-19 experienced profound upheaval in their usual daily rhythm. This study revealed that there was a paradox of experiences consistently shared through their own stories including perceptions healthcare workers had of their leadership and the perceived system failure. *Implications for Nursing Management*. The pandemic resulted in healthcare teams expressing anxiety in caring for COVID patients, yet trying to remain hopeful. They also expressed awareness of how their own roles changed and the leadership failures during this time. Strong leadership is required to create a path forward for healthcare workers.

## 1. Introduction

The experiences during the COVID-19 pandemic, over the past two years, have resulted in an incomprehensible impact on everyday life. Everyone has personal COVID-19 experiences and memories, and none are more vivid than those of healthcare workers. As the time passes, memories may fade, yet stories will remain. It would be wise to examine the stories shared by healthcare workers as a reminder of what happened and what we can learn from those experiences.

Storytelling has demonstrated benefits as an effective tool to educate, build self-efficacy, strengthen coping strategies, and process adverse life events [1]. Telling stories is one of the most powerful means we have as a culture to document, share, learn, and promote connections. The use of digital audio storytelling is becoming more common as a tool to use in health promotion and wellness [1–3].

Everyone has a story. Yet, the literature is lacking in how the stories from healthcare workers during the pandemic affected the individual healthcare worker at that time or how these stories are embedded in patient care and healthcare systems. The purpose of this article is to explore the results from a qualitative analysis of audio stories captured during the second wave of the COVID-19 pandemic and second to compare these results to a prior analysis of audio stories captured during the first wave of the COVID-19 pandemic. These digital stories are one facet of a larger study conducted during the first year of the pandemic. Examining this data collected over time will add to the identification and support needed to change within the healthcare system.

## 2. Methods

### 2.1. Design, Setting, and Study Population

**Figure 1:**
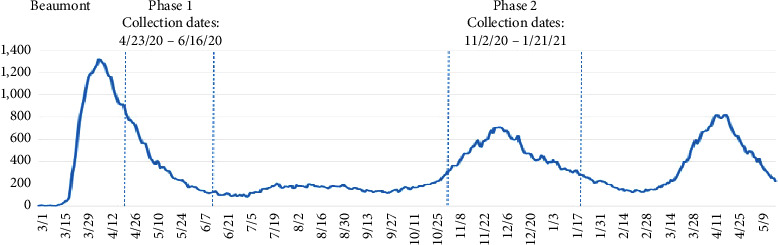
Timeline of data collection correlated with COVID-19 cases in the health system.

A descriptive qualitative study was conducted within a large healthcare system in Southeast Michigan. Data collection began in April 2020 through January 2021. Audio diaries were collected during the first and second waves of the pandemic in Michigan, as measured by the number of patients hospitalized with COVID-19 across the study healthcare system (see Figure 1). The second wave analysis focused on stories collected from November 11, 2020, to January 21, 2021 (see Figure 1). Recruitment targeted all healthcare workers, not just those on the frontline or involved in direct patient care. Invitations to participate were periodically included in daily COVID-19 update emails sent out by the health system's communication department to all employees and contracted providers.

### 2.2. Data Collection

The recruitment announcements directed interested participants to a study website, where they could consent to participate in the study, provide basic demographic information, and submit an audio recording. The consent form included the following statement: *you can talk about whatever you would like to share. This might include stories about your own experience, stress, or emotional response; observations regarding the health system or individual preparedness, responsiveness, or adaptation regarding the pandemic; or anything regarding individual or collective ability to care for our patients and ourselves.* There were no specific prompts once they completed demographic data collection and proceeded to the recording webpage. A list of support resources was provided in case participants were in distress. Participants were asked to not include any personal or patient identifiers in their recordings. The audio recording was limited to 5 minutes. Participants had the option to return to the website and record as often as they desired.

### 2.3. Data Analysis

Each story was recorded using Camera Tag^®^ which included both the audio recording and corresponding written transcription. A grounded theory approach was used to analyze the audio diary content, and both audio recordings and written transcripts were analyzed concurrently. Two reviewers, using an open-coding method, created a line-by-line analysis. After their independent review, a comparative iterative process was used to synthesize results and refine the themes. A third reviewer examined the results for further refinement, clarity, and reduction of bias [4]. In addition, in order to minimize investigator biases, researchers coding the second wave of audio stories were unaware of the results of the analysis of the first wave of stories. Once the second wave analysis was carried out, results were reviewed by both teams, and similarities and differences in outcomes using a constant comparative process were identified.

### 2.4. Ethics Approval

Study participation was anonymous and voluntary. Ethical approval was obtained by the institutional review board.

## 3. Results

### 3.1. Demographics

There were twenty-one audio stories submitted between November 11, 2020, and January 21, 2021. The demographic and participant characteristics captured were age, gender, and role in patient care (see Table 1). The participants ranged in age from 18 to 74 years old, with approximately one-half (54.2%) under 44 years old. Interestingly, there was 23.8% of respondents in the 55–64-year age range and 9.5% in the 65–74-year age range. These groups from 55 years and over are approaching retirement age yet were working during this time. More women (61.9%) participated than men (38.1%).

Also, lastly, 71.3% of participants stated they were involved in some area of direct patient care: 19% a medical provider, 19% nursing, and 33.3% in the other healthcare provider category. This also included 19% in administrative roles. Many of the audio diaries included the roles participants were engaged in during their recordings.

### 3.2. Audio Stories

The qualitative analysis revealed four themes. They were as follows: negative emotional response/impact, leadership/administrative failures, imposed or changing role expectations, and concerned feelings/statements of optimism and hope. Findings are summarized in Table 2. Their stories revealed a rich tapestry of experiences, feelings, interpretations, and actions needed to be taken and how they adapted to the COVID-19 pandemic. The following paragraphs display the range of statements found in each theme.

### 3.3. Negative Emotional Response/Impact

Statements describing the intense emotions related to living and working in health care during the pandemic were found in this theme. Fifteen of the twenty-one diaries (71.4%) included words, phrases, and audible emotions. Statements from participants such as “it was a total nightmare” (diary #1), “most difficult time of my life” (diary #14), “it is stressful, every day is still stressful” (diary #10), and “lots of anxiety” (diaries #2, 5, 10, 12, and 16) were present. Audible emotions, such as deep sighing, pausing, and even being obviously choked up, were heard. The following direct statements are included:

“*I don't think anyone who has not actually worked for 12 hours …with COVID patients can understand, not administrators, not supervisors. It was unreal, it started with the shutdown when we told not to wear masks…, “I would cry every single day”* (diary #5).

Another participant stated:

*“this has just been really, really hard. People are dying every day; all the time and we're supposed to keep going like nothing is wrong” (diary #11)*

Also, another comment is as follows:

*“in the ICU it was very traumatic period…many times I was the last face people saw and I held their hand and talked to them before they were intubated…many of them did not survive” (diary#15).*

These are examples of the feelings from healthcare workers, from their own lens, as they lived and worked during the COVID-19 crisis all the while trying to do their best.

### 3.4. Leadership/Administrative Failures and Concerns

The leadership theme captures the words and phrases regarding leadership responses 8–11 months after the onset of the pandemic. Seven diaries (33.3%) contained words or phrases expressing their thoughts on how leadership responded. Participants used phrases such as “mixed messages” (diary #5), “leadership response was a disgrace” (diary #9), “felt dissociated from administration” (diary #15), “seemed to be in the bunker” (diary #15), and “healthcare hero did not include all staff” (diary #10) as well as “felt hospital did the best they could” (diary #5).

The deeper stories told a tale of negative perceptions towards healthcare leadership and administration. One participant shared:

*“the response of the hospital has been a disgrace. They have done nothing to keep the staff, the patient's, the doctors or providers safe. They've made this terrible pandemic worse in every way and fashion. We have to beg for the ability to get protective equipment. They've provided no universal testing, no clear guidance on how to deal with colleagues that are positive, and limited support, I'm completely disappointed” (diary #9).*

Also, another stated:

*“I felt very disassociated from administration because they seem to be in a bunker and put out information that oftentimes was false and misleading. Unfortunately, the anesthesia contract was canceled during COVID which made for really poor morale. Because of the administration's actions the environment has become more and more toxic period. It's an unfortunate period and believe I will have PTSD as a result of this experience” (diary #15).*

Also,

*“There were mixed messages from the administration about when we were supposed to work and when we were to stay home…. I do not hate my hospital but the fallout always ended on the nurses” (diary #5).*

While another stated:

*“We didn't get healthcare hero support on the nightshift. We were basically ignored… everything about the nightshift was forgotten...and it really hurt morale” (diary #10).*

These are the stories of how participants perceived leadership's failure to communicate effectively and support all healthcare workers. Healthcare workers felt that they bore the brunt of care and that there was an expectation of something more from their leadership.

### 3.5. Imposed or Changing Role Expectations

These statements uncover the awareness of the participants on how their own roles changed due to the pandemic. Some were deployed from their home area to other areas in the hospital, and others were asked to do other roles beyond their usual assignments. More were in usual roles with new or added responsibilities, and lastly, some were working remotely. There was an overall awareness of how their day-to-day lives, regardless of whether they worked in direct care or indirect care even including home life, changed. Seven of the diaries (33.3%) included statements that reflected an awareness of how their roles changed. The following displays the actual stories shared:

*“Many of my departments were redeployed to bedside care, um, none of us having clinical care expertise um, in many years…. we didn't feel we could provide safe patient care…. We could assist the primary RN as much as possible during a very difficult time…. We feel there are many ways we could help including being family liaisons or just RN helpers.. that is my experience, um..thank you” (diary #2).*

Also,

*“I volunteered for a COVID unit and it definitely brought us together as a work family. I volunteered on a unit I hadn't worked before and it didn't take long for them to make me, include me like family on the team…they were happy to have me” (diary #14).*

Also, other role changes resulted from roles outside of the hospital. One story included

*“I worked in the trades is very stressful because we did cut at this facility and it was hard and I had to do a lot of extra work at home to support my wife” (diary #21).*

These statements all share how healthcare workers were aware of the significant changes in work roles, work assignments, and balancing home life needs.

### 3.6. Feelings/Statements of Optimism and Hope

These were statements related to how their experiences had some measure of goodness. Their stories revealed descriptions of what they learned, pride in how they coped, and positive personal outcomes. Acknowledging the magnitude of these moments, there were statements of hope for the future and optimism for positive outcomes. Nine of the diaries (42.8%) included statements related to hope and optimism. Words of hope related to vaccine development and maintaining positive personal outlook were found. Specific statements are as follows:

*“With the second surge, I think I am more confident now, not like before, being afraid. Thank God there is now a vaccine out. Let's hope that the vaccine will work” (diary #13)*

Other stories included statements, such as

*“I feel very lucky to have had the COVID, experience it's been an interesting time in history… I really liked being at home with my kids more than in the past. I feel like before COVID came around I was having a hard time with my work life balance where I was spending you know much more energy on working than I was on my family” (diary #7),*

Also, lastly,

*“I got to learn how to wear special equipment...that would keep me safe …it gave me a chance to try new things” (diary #6)*

*“I know our lives are changed but I hope we can be better people for this…I am hoping 2021 will be a better year” (diary #19)*

*“there is more hope right now since the vaccine is available” (diary #13).*

During these remarkable times, maintaining hope and optimism remains. These stories told us while many recognized the horror of caring for people during the COVID-19 pandemic, they were still able to look forward seeking a better tomorrow.

## 4. Discussion

These stories revealed the complex, multifaceted, and emotional response of healthcare workers in a wide variety of roles. Each audio diary contained at least one to three of the above mentioned identified four themes.

Comparing the first and second audio diary analyses from the larger study offered the following insights [5]: First, there was a continuation of stories relating to both fear and hope. The first analysis labeled the themes as paradoxical. Paradoxical Theme 1: harsh work environment resulted in psychological distress, contrasting with personally rewarding experiences which aligned with negative emotional response/impact and feelings/statements of optimism and hope. It is unclear at this point what conclusions can be drawn from these results. One might suggest that each day brought a new challenge with no clear endpoint. Yet, it certainly demonstrates the deep impact of their perceptions, interpretations, and memories of living through a pandemic. Giving voice to one's experiences, finding meaningful purpose, and maintaining hope during critical times in one's life are an essential coping strategy [6–8].

While the first two themes appeared in the previous analysis during the first wave of the pandemic [5], the themes of leadership failures and changing roles emerged during the second surge of the pandemic. Though many studies also reported similar findings related to the first two themes, relatively few studies have highlighted leadership issues and changing role expectations during the pandemic [9, 10]. It might be purported that it was so early in during the crisis that the priorities of care left little time for leadership to fully understand the role needed to lead and support the staff. As COVID-19 unfolded, the information and directions changed constantly as infections spread and death rates climbed. The immediate focus was on PPE, staffing, isolation, or standards of care, and that was the leadership focus. The staff's need for direction and support followed the awareness of changing roles as care needed to be adapted, staffing issues became more apparent, and resources were changed.

Leadership failures/concerns were not an expected result. Comments ranging from no clear guidance or mixed messages are overshadowed with more severe comments of “disgrace” to “toxic workplace.” During times of crisis, people look to their leaders for direction, meaning making, and/or empathy [11, 12]. These results revealed there was a perceived lack of direction, meaning making, and empathy displayed by their healthcare leader. For healthcare workers, these stories acknowledge the intense uncertainty that persisted, and yet, they still expected leadership to safely guide them.

The literature refers to these unprecedented situations as “wicked problems” [13]. Wicked problems are defined as those problems with a social or cultural context that is difficult or impossible to solve for as many as four reasons: incomplete or contradictory knowledge, the number of people and opinions involved, the large economic burden, and the interconnected nature of these problems with other problems [14]. Clearly, healthcare workers perceived that their leadership failed in supporting their work as they were presented with many “wicked problems” from supply shortages, staffing, and a huge loss of life. It is not surprising that workers were confused by the day-to-day changes made in protocols, staffing, and processes or the challenges of keeping up with communication in a large system. Nonetheless, leadership must acknowledge these perceptions and address them up front to renew faith with their workers.

### 4.1. The Benefits of Capturing Stories

The fallout from working during these times has resulted in poor morale, changes in quality standards, severe staffing shortages, and morale distress [10]. It is not about what is right or wrong but what comes next. Now is the time for leadership to take a breath and lean into addressing the concerns of staff so that the system can recalibrate towards a postpandemic world [15].

These audio diaries may have provided some therapeutic benefits for the staff responding by being able to put their thoughts into words and “get it out.” Using the stories directly from healthcare workers opens an intimate lens to their experience “through their own eyes.” From the researcher's perspective, hearing the audio recordings while reading the transcripts also offered the ability to witness the participant's emotional prosody, the nonverbal tone of the speech, which includes loudness, speech rate, and pauses. Hearing the vast array of emotions added depth and clarity. Stories are powerful. Using stories, Bennett el al. [9] explored the impact of the pandemic on healthcare workers with similar results. Their study was conducted within the first wave of the pandemic and also identified the disconnection between leadership and frontline staff. Other studies using stories during this time reported comparable themes related to emotional response, hopelessness, and hopefulness but no leadership concerns [9]. Furthermore, there is a belief that being able to articulate their own experiences with limited structure or judgement may be therapeutic to the storyteller [1].

Understanding healthcare workers stories today will allow leaders to address the concerns of healthcare workers for the future. The first step starts with the awareness of the issues. While these audio dairies captured a moment in difficult times, the awareness of the leadership failures correlates with the awareness of role change from frontline workers. Their stories told us that the staff did not feel their leaders were present, gave clear messages, or displayed empathy. Protocols were changed rapidly, and social isolation contributed to the fragmented communication. Simply stated, healthcare workers' stories reveal not only a failure of leadership but also of an acute need for effective leadership. It is essential for leaders to use all tools available to address these issues as avoiding it will only increase the anguish of the lived experience from the pandemic [16]. Clearly, now would be a time for leaders to reflect on what worked and what needs improvement.

### 4.2. Limitations

The study's limitations include a relatively small convenience sample from a single large healthcare system in the Midwest United States. The sample size represented a wide range of healthcare positions, from intensive care staff to administrative roles. It is unclear how various roles differed in their experiences during the pandemic. It may only represent people who had a particularly distressing or rewarding experience. Respondents represented those who were comfortable and able to use the audio recording, and recording could be carried out in any location with an Internet connection. It is also unclear where and when the respondents chose to record their stories and how this may have influenced their responses. Participants were able to sign in to recording their stories as many times as they wished; yet, no one signed in more than once or used the entire five minutes for their story. Generalizability is limited.

### 4.3. Implications for Nurse Leaders

The audio diary study represents a small snapshot into the daily lives of healthcare workers captured through their own stories during the second surge of the pandemic. As the pandemic unfolded, understanding the changes in lived experiences allows for a better response in preparation should another healthcare crisis, however unlikely, occur. The use of storytelling is a valuable tool to capture real-time thoughts, perceptions, and practices [17]. It allows for connection through discussions of shared experiences and adds to the growing body of knowledge, documenting the fear and emotional toll for healthcare workers, identified avenues for hope, and finally clearly revealed acute care leadership shortcomings [3]. Further research is needed as healthcare continues to address the multitude of the COVID-19 issues challenging healthcare systems.

While no one could have predicted a pandemic, not addressing the resultant fallout would be reckless. The outcomes of the COVID-19 pandemic are complex and complicated. Addressing the impact from COVID-19 goes beyond developing new protocols or treatments. The postpandemic healthcare world has created a cultural shift. One example is the severe nurse staffing shortage. Nurses remained at the frontline of caring for the very ill and were considered heroes by the public. Yet, the vaccine mandates forced nurses to resign, adding to the many other reason's nurses left the bedside [6]. New technology filled the gap as healthcare workers were limiting their exposure to infected patients, allowing for the telemedicine practice to emerge [18]. In regard to leadership, the current healthcare research and commentary is filled with suggestions on how leaders can motivate workers, how to recognize intent to leave, and negative perceptions about work place culture [19]. Carruci and Hogan [19] state that having meaningful conversations and focusing on priorities not on productivities are strategies that leaders can use to rebuild their teams. How does this relate to the audio diary study? Sharing stories can create connection. Looking forward to solutions for today's problems, storytelling, and shared experiences can create the opportunity for meaningful conversations, identifying priorities, and rebuilding teams.

## 5. Conclusion

While this is a small study, the results of this study are consistent with those of other studies that were conducted during this time [9, 17, 20, 21]. As the COVID-19 pandemic moves into an endemic phrase, healthcare systems, leaders, and staff will begin to take a breath and begin the process of recovery, from a system perspective and a human perspective. These stories will serve as a powerful reminder as memories may fade and open up a new viewpoint for rebuilding. It is our hope that storytelling will become a valuable tool to increase understanding, process improvement, and hopefully healing.

## Figures and Tables

**Table 1:** Characteristics of the participants.

	Count	%
Number of participants	21	100.0
*Age*		
18–24 years	1	4.8
25–34 years	5	23.8
35–44 years	5	23.8
45–54 years	3	14.3
55–64 years	5	23.8
65–74 years	2	9.5
*Gender*		
Male	8	38.1
Female	13	61.9
Gender other	0	0.0
Involved in patient care	13	61.9
*Role*		
Administrative support services	2	9.5
Administrator/manager	2	9.5
Clerical	1	4.8
Clinical support services	1	4.8
Medical provider	4	19.0
Nurse	4	19.0
Other	4	19.0
Other healthcare provider	3	14.3

**Table 2:** COVID hero audio diary theme descriptors.

*COVID hero audio diary theme descriptors*	
Themes	Descriptors
*Negative emotional response/impact* related to living through and with the pandemic. These were found in the following words, phrases, and audible emotions Overall summary is that those closest to bedside care described (experienced) far more emotional, scary, fearful comments. The audible emotions were apparent in tone, words, and language	(i) “It was a total nightmare”
	(ii) Audible sighing and deep breathing as they told their stories
	(iii) “Most difficult time of my life”
	(iv) Used words such as “anxious,” “PTSD,” “depression,” “increased stress, anxiety, and depression”; only frontline workers can truly understand what we went through
	(v) Fear of unknown (related to changing PPE requirements, begging for PPE, and what happens if they get sick (must use own PTO time))
	(vi) Actions required to be safe from the virus. Protecting self, the best they could
	(vii) Cautious of interacting with others
	(viii) Fear of getting others sick
	(ix) Feelings/awareness of isolation
	(x) Separation from family (sent children to live elsewhere)
	(xi) Developing habits to minimize risk to family (taking clothes off in garage, showering before joining family).
	(xii) Overall awareness that there is much stress, and it has led to many mental health issues
	(xiii) Loss of control led to feelings of hopelessness
	(xiv) “Hardest thing is isolation; can't combat because we can't come together”

*Leadership/administration failures and concerns*. These are statements surrounding how healthcare workers felt their leadership was guiding this crisis. This demonstrates a gap in leadership action and staff perceptions. The following are examples of this theme	(i) Leadership was a “disgrace” or “disappointment”
	(ii) Leaders lacked the ability to keeping people safe
	(iii) No clear guidance
	(iv) “Toxic” workplace
	(v) Financial implications due to lack of bonus checks and lack of hazard pay
	(vi) Felt dissociation from administration, they would go to their “bunkers” and not be present for staff
	(vii) Mixed messages from administration
	(viii) Felt hospital did their best but did not anticipate the fallout
	(ix) Felt frontline managers were transparent and supportive, but upper management was mostly absent

*Imposed or changing role expectations*. These are statements voiced of how their roles changed due to the pandemic. Some were deployed, some were put in other roles, some were in usual roles with new or added responsibilities, dealing with variable staffing issues, and lastly, some were working remotely. There was an overall awareness of how the work has changed. Sense of and description of the adaptation to how their roles changed	(i) Assigned to COVID unit either volunteered or administration assignment. “I volunteered for a COVID unit”
	(ii) ‘I believe there will be more deployments
	(iii) Frontline nurses felt like they bore the brunt of caring for COVID patients. They were expected to fill in
	(iv) Awareness of adaptation needed to perform new roles
	(v) Learning new skills
	(vi) Moving to new units (overlap with positive teamwork)
	(vii) Being put in roles/jobs they did not feel very comfortable or competent
	(viii) Changing policies and procedures
	(ix) Increased volume of care needs

*Feelings/statements of optimism and hope.* These were statements related to how this experience impacted the individual personally. There were comments of what they learned, pride in how they coped, and personal outcomes from going through this struggle. Acknowledging the magnitude of these moments, there were statements of hope for the future and optimism for positive outcomes	(i) Hope for vaccine
	(ii) “Staying positive and focused”
	(iii) “More confident in knowing what to do next time” “I hope it will be better next time (deployments)”
	(iv) Stated awareness of opportunities the COVID-19 pandemic offered
	(v) Adaptation to new roles they would not have had normally
	(vi) Grateful to be able to work remotely
	(1) More time with family, able to catch up with other work due to changing time demands of role
	(vii) Stated it brought out the best in many
	(viii) Statements related to their own improvement over the struggle. Overall gratitude of being able to help. Willingness to help (in any capacity, such as “helpers,” changing units, and changing shifts)
	(ix) Stated it brought out the worst in many

Other comments of relevance	“Don't feel like a hero” (stated multiple times). “There are heroes everywhere” Underlying staffing issues embedded in the stories. Staffing issues related to vacancies, put in roles not truly qualified for, or lack of support staff

## Data Availability

The audio diary data used to support the findings of this study are available from the corresponding author upon request.
